# Tablet, Web-Based, or Paper Questionnaires for Measuring Anxiety in Patients Suspected of Breast Cancer: Patients' Preferences and Quality of Collected Data

**DOI:** 10.2196/jmir.3578

**Published:** 2014-10-31

**Authors:** Maarten W Barentsz, Hester Wessels, Paul J van Diest, Ruud M Pijnappel, Cees Haaring, Carmen C van der Pol, Arjen J Witkamp, Maurice A van den Bosch, Helena M Verkooijen

**Affiliations:** ^1^University Medical Center UtrechtUtrechtNetherlands

**Keywords:** breast cancer, electronic questionnaires, paper questionnaires, quality of collected data

## Abstract

**Background:**

Electronic applications are increasingly being used in hospitals for numerous purposes.

**Objective:**

Our aim was to assess differences in the characteristics of patients who choose paper versus electronic questionnaires and to evaluate the data quality of both approaches.

**Methods:**

Between October 2012 and June 2013, 136 patients participated in a study on diagnosis-induced stress and anxiety. Patients were asked to fill out questionnaires at six different moments during the diagnostic phase. They were given the opportunity to fill out the questionnaires on paper or electronically (a combination of tablet and Web-based questionnaires). Demographic characteristics and completeness of returned data were compared between groups.

**Results:**

Nearly two-thirds of patients (88/136, 64.7%) chose to fill out the questionnaires on paper, and just over a third (48/136, 35.3%) preferred the electronic option. Patients choosing electronic questionnaires were significantly younger (mean 47.3 years vs mean 53.5 in the paper group, *P*=.01) and higher educated (*P*=.004). There was significantly more missing information (ie, at least one question not answered) in the paper group during the diagnostic day compared to the electronic group (using a tablet) (28/88 vs 1/48, *P*<.001). However, in the week after the diagnostic day, missing information was significantly higher in the electronic group (Web-based questionnaires) compared to the paper group (41/48 vs 38/88, *P*<.001).

**Conclusions:**

Younger patients and patients with a higher level of education have a preference towards filling out questionnaires electronically. In the hospital, a tablet is an excellent medium for patients to fill out questionnaires with very little missing information. However, for filling out questionnaires at home, paper questionnaires resulted in a better response than Web-based questionnaires.

## Introduction

With the evolution of modern technology, electronic applications are increasingly being used in hospitals. Web-based applications and touchscreen devices are finding their way into hospitals for numerous purposes. These electronic applications can be useful for research purposes, for collecting patient-reported outcomes, and questionnaires [1-3]. Some of the most important advantages of electronic over paper questionnaires include easy usage and immediate electronic storage of results. The use of electronic applications has been evaluated for informed consenting procedures, assessing quality of life, medical education, interventions, diagnostics, and filling out questionnaires [1,3-11].

Obtaining high response rates without missing information is important for research purposes, as non-responders can bias study results [12]. Response rates have been found to be lower using electronic questionnaires compared to paper questionnaires [13-15]. In order to potentially improve response rates, specific patient subgroups with a preference for electronic questionnaires could be identified. For example, elderly patients may not be as experienced with electronic applications. Aiello et al compared the use of a tablet to paper questionnaires in a mammography clinic. They found that older women (>60 years) had a slightly harder time learning to use the tablet compared to younger patients, but preference towards the tablet was similar in both groups [2].

The aim of our study was to assess differences in demographic characteristics of patients choosing paper versus electronic questionnaires and to evaluate data quality and completeness of data of both approaches.

## Methods

### Study Context

This study was performed in the University Medical Center Utrecht, the Netherlands (approximate caseload of 180 newly diagnosed breast cancer patients per year). In 2011, same-day diagnosis for breast cancer was introduced with the aim to provide a definitive diagnosis within one day in over 80% of patients. Reducing the time of uncertainty about a diagnosis could potentially reduce anxiety and stress. All patients suspected of breast cancer visited the outpatient breast clinic and underwent physical examination, diagnostic imaging (mammography and ultrasound) with a histological biopsy if indicated, and received a final diagnosis at the end of the day after a multidisciplinary meeting.

**Figure 1:**
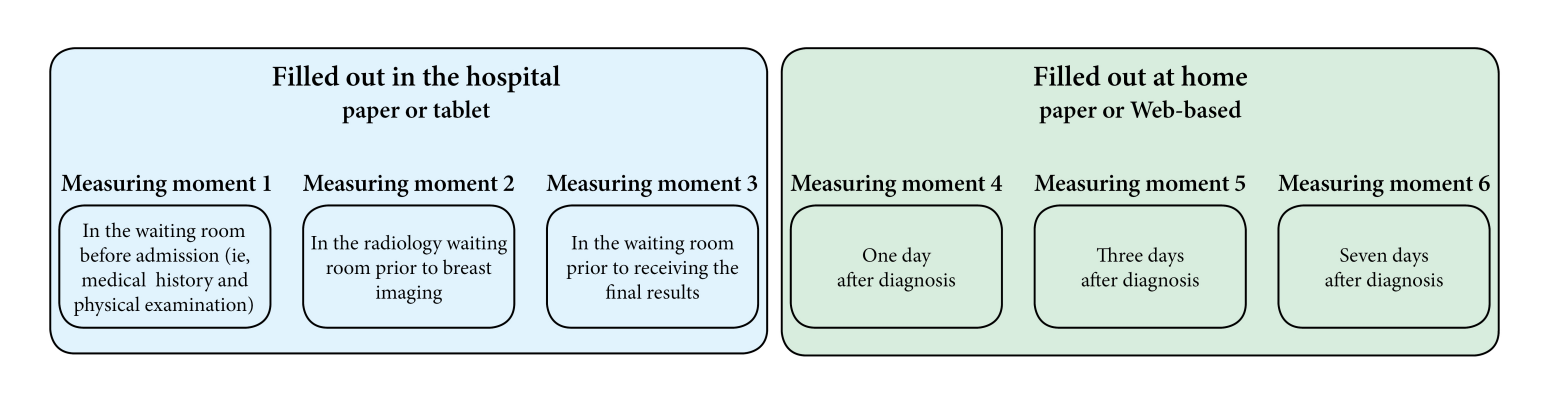
Overview of the six measuring moments during the diagnostic phase to evaluate level of stress and anxiety.

**Figure 2:**
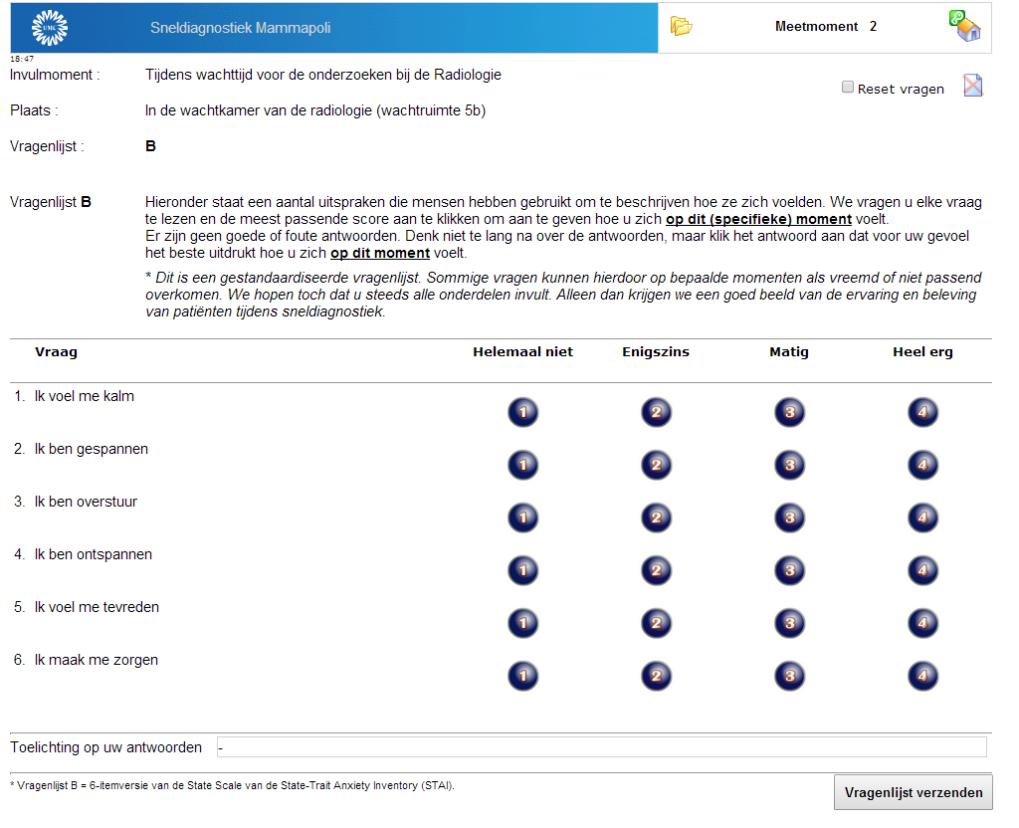
Screenshot of the electronic questionnaire (measuring moment 2).

**Figure 3:**
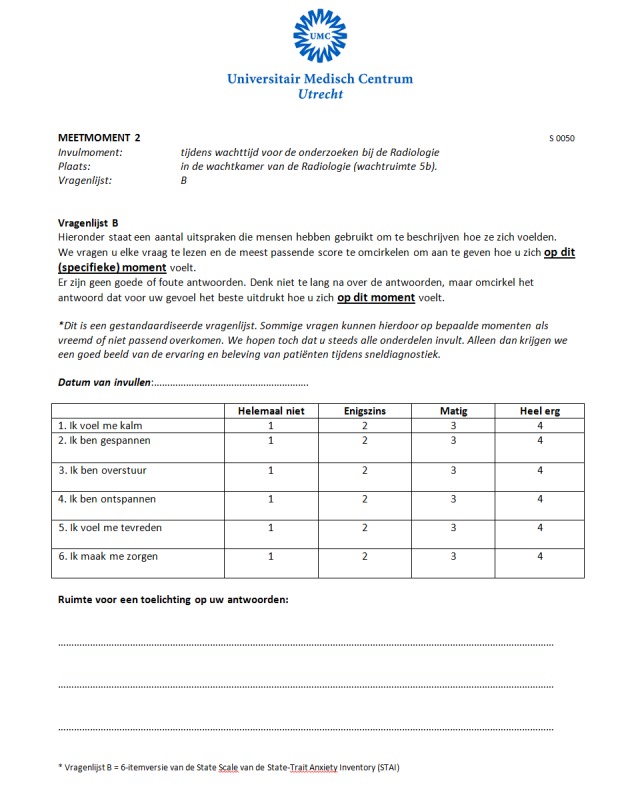
Screenshot of the paper questionnaire (measuring moment 2).

Between mid-October 2012 and June 2013, all patients referred to the same-day diagnosis out-patient breast clinic were eligible to participate in the study. Approval for this study was obtained from the local ethics committee, and all patients signed written informed consent. All patients were asked to fill out the 6-item State Trait Anxiety Inventory (STAI) [16,17] questionnaire at six different time points (measuring moments) during the diagnostic phase to evaluate levels of stress and anxiety (Figure 1). Patients were given the opportunity to fill out the questionnaires electronically (Figure 2) or on paper (Figure 3). Preference towards paper or electronic questionnaires was measured at baseline. The paper questionnaires were returned by mail. In the electronic scenario, the first three questionnaires (administered on the day of diagnosis in the hospital) were offered by means of tablets (iPad). For the last three electronic questionnaires that were to be filled out at home, we used Web-based (hypertext markup language [HTML]) questionnaires. An email with login information to the questionnaires was sent to participants by email on the diagnostic day. The STAI questionnaire was displayed on one page, and all six questions needed to be answered before the form could be submitted. If a question was left blank, an automated message appeared saying that all questions needed to be answered. Patients were not able to look back at previously completed questionnaires. The tablets were also used for providing information and entertainment. An information app was built to provide information on the diagnostic process, diagnostic procedures, treatment team, and routing during the diagnostic day. Several forms of entertainment were available on the tablet, including digital newspapers, magazines, games, and music. The paper questionnaires were returned by mail in a pre-stamped return envelope.

### Outcome Measures

Outcome measures included differences in demographic characteristics between patients choosing paper or electronic questionnaires and data quality, focusing on age, reason for referral, breast cancer history, level of education, and baseline anxiety. Data quality was assessed by focusing on missing information, defined as a questionnaire containing at least one unanswered question. To assess if a breast cancer diagnosis affected the quality of data, subgroup analysis including only patients with a benign diagnosis was performed.

### Methods for Data Analysis

Demographics, history of breast disease, and diagnostic findings were described as proportions and means with standard deviation. Differences in demographic characteristics, reported anxiety score, and completeness of reported data between the electronic and the paper group were compared by means of chi-square test and independent samples *t* test, where appropriate. Significant differences were defined as *P* values of .05 or less. All statistical analyses were performed using SPSS version 20.0.

## Results

### Demographic Data

Of 321 patients referred to our out-patient breast clinic, 136 patients (42.4%) agreed to participate in the study. All patients were offered the choice of paper or electronic questionnaires.

The mean age was 51.3 years (range 18-85 years) and 35.3% (48/136) patients chose to fill out the questionnaires electronically (Table 1). Reason for referral, family history of breast cancer, and breast-related medical history were similar in both groups. Baseline anxiety scores (as measured by the STAI) did not differ between the groups (46.4 in the paper group versus 43.8 in the electronic group, *P=*.30). Diagnostic imaging findings and proportion of patients undergoing biopsy were similar in both groups. Patients choosing to fill out questionnaires electronically were significantly younger compared to those opting for paper questionnaires (47.3 years vs 53.5, respectively; *P=*.01) and had a higher level of education (*P=*.004).

### Outcome Data: Missing Information

There was significantly more missing information (ie, questionnaires containing at least one unanswered question) in the paper group during the diagnostic day (measuring moments 1-3) compared to the electronic group (28/88 vs 1/48, *P*<.001) (Table 2). In the paper group, this included two patients who did not fill out one or two questions (instead of complete questionnaires not filled out).

In the week after the diagnostic day (measuring moments 4-6), missing information was significantly more prevalent in the electronic group (41/48, 85%) compared to the paper group (38/88, 43%) (*P*<.001). This included 7 patients in the paper group who left one or two questions unanswered. These differences persisted in subgroup analysis including only patients with a benign diagnosis.

**Table 1:** Demographic characteristics of patients undergoing 1-day diagnosis for suspected breast cancer, comparing patients choosing paper questionnaires (n=88) to those choosing electronic questionnaires (n=48).

Characteristics		Paper, n (%)	Electronic, n (%)	*P* value^a^
Female		86 (98)	47 (98)	.94^b^
Age in years, mean (SD), range		53.5 (14.1), 22-85	47.3 (11.4), 18-66	.01^c^
**Reason for referral**				
	Screening	27 (31)	16 (37)	.75^b^
	Palpable lesion/symptoms	61 (69)	32 (67)	
Positive family history of breast cancer – yes		34 (39)	13 (27)	.18^b^
Previous breast disease/complaints – yes		28 (32)	19 (40)	.36^b^
Previous breast cancer diagnosis – yes		4 (5)	3 (6)	.67 ^b^
**Breast Imaging Reporting and Data System (BI-RADS) classification suspected lesion**				
	I	18 (21)	9 (19)	.54^b^
	II	34 (39)	22 (46)	
	III	9 (10)	9 (19)	
	IV	15 (17)	5 (10)	
	V	10 (11)	3 (6)	
	VI	1 (1)	0 (0)	
	No imaging performed	1 (1)	0 (0)	
**Biopsy performed**				
	No	51 (58)	29 (60)	.62^b^
	Yes, histology	32 (36)	18 (38)	
	Yes, cytology	5 (6)	1 (2)	
Cancer – yes		16 (18)	7 (15)	.59^b^
**Level of education** ^d^				
	Low-moderate^e^	21 (26)	1 (3)	.004^b^
	Moderate-high^f^	27 (33)	10 (29)	
	High^g^	33 (41)	24 (69)	
	Missing	7	13	
Mean baseline anxiety score, STAI (SD)		46.4 (13.3)	43.8 (13.5)	.30^c^

^a^ *P* values are based on valid proportions.

^b^Calculated by chi-square test.

^c^Calculated by independent samples *t* test.

^d^Level of education is based on the Dutch educational system

^e^Low-moderate education includes primary education/low pre-vocational/secondary general education.

^f^Moderate-high education includes secondary vocational/higher general and pre-university education.

^g^High education includes higher vocational education/university.

**Table 2:** Differences in proportion of patients with incompletely filled out questionnaires between patients opting for paper questionnaires (n=88) and patients choosing electronic questionnaires (n=48).

	Paper, n (%)	Electronic, n (%)	*P* value
Baseline – moment 1 – STAI 1	25 (28)	0 (0)	
Measuring moment 2 – STAI 2	24 (27)	1 (2)	
Measuring moment 3 – STAI 3	25 (28)	1 (2)	
Measuring moment 4 – STAI 4	28 (32)	27 (56)	
Measuring moment 5 – STAI 5	31 (35)	34 (71)	
Measuring moment 6 – STAI 6	35 (40)	33 (69)	
Measuring moment 1-3 – in hospital^a^	28 (32)	1 (2)	<.001
Measuring moment 4-6 – at home^b^	38 (43)	41 (85)	<.001

^a^Includes all patients with at least 1 incomplete questionnaire in measuring moment 1, 2, or 3.

^b^Includes all patients with at least 1 incomplete questionnaire in measuring moment 4, 5, or 6.

## Discussion

### Principal Findings

The use of tablets and Web-based questionnaires for collection of patient-reported data has many potential advantages over the use of paper questionnaires. Still, the present study shows that a majority of patients preferred paper over electronic questionnaires. Younger patients and those with a higher level of education were more likely to opt for electronic questionnaires. When using tablets (during the diagnostic day in the hospital), more complete information was collected compared to using paper questionnaires. These data suggests that tablets are superior to paper questionnaires. However, the use of Web-based questionnaires resulted in less complete data collection than paper questionnaires. This might be due to the study design where patients could fill out the electronic questionnaires only on a specific day.

A major advantage of filling out electronic questionnaires is that information is immediately saved. Other advantages of the use of tablets include the possibility of automatically reminding the patient to fill out the questionnaires, and providing information and entertainment. We did not electronically remind patients by email to fill out the questionnaires. Considering the high percentage of incompletely filled out Web-based questionnaires (85%), we would definitively incorporate this in a future study. We did use an automated message when not all questions were answered. This resulted in completely filled out questionnaires in the electronic group, which could possibly lead to more complete data.

Possible drawbacks of using Web-based questionnaires are high non-response rates, impaired reliability and validity, and safety or confidentiality issues [18]. Drawbacks of tablets are the need for upgrades, wireless network unreliability, hardware theft [19], and costs. Fritz et al performed a cost-effectiveness analysis comparing the costs of electronic questionnaires offered on a tablet with paper questionnaires. They found the break-even point to be at 1737 paper sheets per year [1].

Completeness of data collection was very high in the tablet group, with only 1 of the 48 patients not filling out all questions at the first three measuring moments. Missing information was highest in the Web-based group, where many patients (41/48) did not fill out all questions at the last three measuring moments. One likely reason for the high rate of missing information in this group was that patients could fill out the questionnaires only on the correct day (ie, exactly 1, 3, or 7 days after the patient’s visit). Our aim was to measure patients’ anxiety at specific moments in time, and we limited the possibility of filling out the questionnaires to the correct day only. Patients in the paper group, however, were able to fill out the questionnaires at any given time. This led to more missing information in the Web-based group, and results on missing information need to be interpreted with this information in mind. However, limiting patients to one specific moment to fill out the questionnaires might lead to more accurate measurements of patients’ anxiety at that specific moment. For higher response in the Web-based group, automated email reminders could be useful.

### Limitations

A possible limitation of this study was that we included only breast cancer patients and consequently, 98% were female. These results are therefore not generalizable to other populations. There could be reduced missing data in the electronic group when other groups are included in a similar study (eg, men, young adults).

### Conclusions

Younger patients and patients with a higher level of education have a preference towards filling out questionnaires electronically. In the hospital, a tablet is an excellent medium for patients to fill out questionnaires with very little missing information. However, for filling out questionnaires at home, paper questionnaires result in a better response compared to Web-based questionnaires.

## Abbreviations

BI-RADS: Breast Imaging Reporting and Data System
HTML: hypertext markup language
STAI: State Trait Anxiety Inventory
